# Lower prefrontal activation during emotion regulation in subjects at ultrahigh risk for psychosis: an fMRI-study

**DOI:** 10.1038/npjschz.2015.26

**Published:** 2015-09-23

**Authors:** Jorien van der Velde, Esther M Opmeer, Edith J Liemburg, Richard Bruggeman, Roeline Nieboer, Lex Wunderink, André Aleman

**Affiliations:** 1 Neuroimaging Center, University of Groningen, University Medical Center Groningen, Groningen, The Netherlands; 2 Academy for social studies, Hanze University of Applied Sciences, Groningen, The Netherlands; 3 Rob Giel Research Center, University Medical Center Groningen, Groningen, The Netherlands; 4 Department of Psychosis studies, Mental Health Care Friesland, Leeuwarden, The Netherlands; 5 Department of Psychology, University of Groningen, Groningen, The Netherlands

## Abstract

**Background:**

Previous research has shown that patients with schizophrenia experience difficulties with emotion regulation and activate prefrontal regions to a lesser extent during reappraisal of emotional information. It has been suggested that problems in emotion regulation might precede the onset of psychosis. Therefore, it could be hypothesized that also individuals at ultrahigh risk (UHR) for developing psychosis experience difficulties with emotion regulation.

**Aims:**

The aim of the current study was to investigate whether individuals at UHR for developing psychosis show abnormal brain activation during reappraisal of negative pictures.

**Methods:**

Using functional magnetic resonance imaging (fMRI), we scanned 15 UHR participants and 16 matched healthy controls while performing an emotion regulation task. During this task, participants had to reappraise their negative emotion elicited by International Affective Picture System pictures. Furthermore, the reported use of reappraisal was examined with the emotion regulation questionnaire (ERQ).

**Results:**

Individuals at UHR for psychosis showed less activation in the left ventrolateral prefrontal cortex during reappraisal compared with healthy controls. Furthermore, they reported less use of reappraisal in daily life (*P*=0.01; 95% CI (0.24–1.63)).

**Conclusions:**

These findings indicate that dysfunctional emotion regulation may already occur in individuals at risk for psychosis. These regulation difficulties are underpinned by less ventrolateral prefrontal cortex activation, and may result in high negative affect, lower social functioning, and high rates of psychotic symptoms.

## Introduction

Although schizophrenia is widely recognized to involve impaired cognition,^1^ research is increasingly uncovering emotional abnormalities as well.^2,3^ For example, previous research has shown that patients with schizophrenia experience high levels of negative affect.^4^ It has been suggested that this increased negative affect precedes psychotic symptoms, such as hallucinations and delusions, due to dysregulation of affect.^5–7^ Emotion regulation can be described as the process of changing the experience and expression of emotions.^8^ Reappraisal is a frequently used emotion regulation strategy that implies a reevaluation of emotional stimuli in such a way that they become less emotionally disturbing.^8^ Behavioral studies have shown that patients with schizophrenia experience difficulties with downregulating negative affect through reappraisal^9^ and tend to use reappraisal less often compared with controls.^10–12^ Not all studies confirmed this latter finding, however.^13–15^

In healthy people, activation of the dorsolateral, dorsomedial, and ventrolateral prefrontal cortex (DLPFC, DMPFC, VLPFC) has been shown to increase during reappraisal.^16^ Subsequently, amygdala activation has been suggested to decrease, resulting in lower negative affect.^16^ However, in patients with schizophrenia less activation has been found in the DLPFC and VLPFC during reappraisal compared with healthy controls.^9,17,18^ Furthermore, the functional connectivity between the PFC and the amygdala has shown to be weaker in patients with schizophrenia.^9^

Interestingly, it has been suggested that emotion regulation difficulties may precede the first onset of psychosis.^5,7^ One way of examining this hypothesis is to investigate whether emotion regulation difficulties are already present in individuals with an At Risk Mental State (ARMS), which often precedes the onset of psychosis. This ARMS is characterized by subclinical psychotic symptoms and a decline in social and global functioning.^19^ Individuals with this ARMS have an ultrahigh risk (UHR) for developing psychosis with transition rates of 29% after 2 years.^20^ Furthermore, UHR individuals report higher levels of distress, negative affect, and lower levels of social functioning.^21,22^ Individuals who do not develop psychosis often still experience psychiatric problems.^23^ Therefore, it is important to gain more knowledge about difficulties in emotion regulation that may already be present in this UHR group. Remarkably, to the best of our knowledge, no studies have yet examined emotion regulation and its neural basis in this group.

The aim of this study was to examine whether brain activation during emotion regulation in UHR individuals differed from healthy controls. Previous research has shown that UHR individuals experience difficulties during emotion processing. Specifically, compromised cognitive-emotional processing^24,25^ and high levels of negative affect^26^ indicate possible difficulties with emotion regulation in this group. We therefore hypothesized reduced recruitment of the emotion regulation circuitry. More specifically, we expected reduced activation in the dorsolateral, ventrolateral, and the dorsomedial prefrontal cortex, because these are widely involved in emotion regulation.^16^ Furthermore, we expected to find higher activation in the amygdala in UHR individuals, due to less downregulation of this region.^16^

## Materials and methods

### Participants

Sixteen participants at UHR for developing psychosis were recruited from a help seeking population from the Mental Health Care Services Friesland, The Netherlands. All participants between 18 and 35 years of age, referred to the Mental Health Care Services Friesland were prescreened with the Prodromal Questionnaire (PQ-16 (ref. 27)). A score of ⩾6 resulted in the administration of the Comprehensive Assessment of At Risk Mental States (CAARMS^28^) by a trained psychologist. It has been suggested that 16 subjects is enough to optimize sensitivity to large effects in functional magnetic resonance imaging (fMRI).^29^

Participants were selected for taking part in the MRI study if: (1) they had a genetic risk for developing schizophrenia or CAARMS-scores in the range of ARMS (as defined in Reitdijk *et al.* ^30 ^); and (2) they had an impairment in social functioning (SOFAS-scores ⩽50 in the last year or a 30% drop in SOFAS scores within one month^31^). This approach is in accordance with the procedure of the EDIE-NL trial.^30^ Exclusion criteria were: (1) a history of psychosis; (2) neurological diseases (participants with headaches were not excluded); and (3) MRI contraindications. The UHR participants were compared with 16 healthy controls without a presence or history of psychiatric or neurological disorders, matched on age, gender, education, and handedness.

All participants gave written informed consent and the study was approved by the Mental Healthcare Research Ethics Committee (METIGG). The UHR participants were scanned within 6 months of the CAARMS assessment. Demographic and clinical characteristics of the final sample (15 UHR and 16 controls, for the reason of exclusion see results) are presented in Table 1.

### Behavioral measurements

The emotion regulation questionnaire (ERQ^32^) was applied to assess the use of the emotion regulation strategies, reappraisal (giving a different meaning to emotional stimuli in such a way that the emotional content decreases), and suppression (the inhibition of emotion–expressive behavior). The ERQ comprises ten items of which six examine reappraisal and four examine suppression. Participants had to rate on a seven-point scale to what extent a certain statement applied to them (strongly disagree–strongly agree). To make the subscales more comparable a relative score was calculated by dividing the total scores on the subscales by the number of items comprising that subscale.

The positive and negative affect scale (PANAS^33,34^) was administered to examine the current affective state. The scale consists of 10 positive items and 10 negative items. Participants had to rate to what extent they experienced certain mood states on a five-point scale.

To examine the clinical characteristics of the UHR individuals, the semi-structured interview Positive and Negative Syndrome Scale (PANSS^35^) was administered on the day of the fMRI scan. In this 30-item interview, positive, negative, and general symptoms of psychosis that occurred in the week before the scan session were measured.

### Emotion regulation task

**Figure 1:**
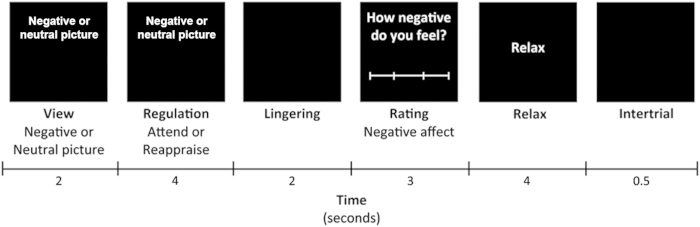
Experimental design for a single trail. Adopted from van der Meer *et al.*^18^

The emotion regulation task (adapted from^36^) consisted of three conditions, attend neutral, attend negative, and reappraise. The stimuli consisted of 44 negative and 22 neutral pictures from the International Affective Picture System. Each trial was constructed as follows (see Figure 1): First, a picture appeared with the instruction to ‘view’ the picture (view phase, 2 s). This View phase was included to examine emotion processing. Subsequently, the word ‘view’ changed in either ‘reappraise’ or ‘attend’ (regulation phase, 4 s), which was included to examine the neural correlates of emotion regulation. During reappraise, participants had to reinterpret the picture or distance themselves from the content in such a way that it became less emotionally disturbing. During attend, participants were instructed to look closely at the picture and not change the way they were feeling. The neutral pictures were always paired with the ‘attend’ instruction. Negative pictures were paired with either reappraise (22 pictures) or attend (22 pictures). Following regulation, a black screen appeared (Linger, 2 s). After that, participants were asked to rate how negative they were feeling on a four-point rating scale (not negative at all-extremely negative; 3 s). Subsequently, the word ‘relax’ appeared, serving as a short rest period (4 s), followed by a black screen (0.5 s) to alert participants that the next trial was coming. One trial lasted for 15.5 s. After 9 or 10 trials, a fixation cross appeared on the screen for 20 s.

To ensure correct application of the reappraisal strategy, a short training was given prior to the fMRI scan. During this training, participants practiced the reappraisal strategy by telling the researchers how they would apply the strategy in response to several negative pictures.

### Data acquisition

MRI data were acquired using a 3.0 Tesla whole body scanner (Philips Intera Achieva, Best, The Netherlands), equipped with an eight-channel SENSE head coil located at the University Medical Center Groningen. The functional images were acquired by a T2-weighted echo producing 37 slices of 3.5-mm thick with no gap. The images were slightly tilted (30°) to prevent artifacts from the nasal cavities. The functional scans were made in the axial plane (TR=2 s; TE=30 s; flip angle (*α*)=70°; FOV=224.0, 129.5, 224.0; in-plane resolution 64×62 pixels; isotropic voxels of 3.5 mm) and were scanned interleaved. The T1-weighted anatomical image (170 slices; isotropic voxels of 1 mm; TR=9 ms; TE=3.54 ms; *α*=8°; FOV=256 mm) was acquired in the bicommissural plane, covering the whole brain.

### Statistical analyses

Behavioral analyses were performed using SPSS20 (SPSS Inc., Chicago, IL, USA). The behavioral data were visually inspected for outliers (<2 s.d.) and normality. Differences between the UHR and control group on age, education level, PANAS and ERQ, were examined using two-sample *t*-tests. *Χ* ^2^-tests were performed to examine differences in gender and handedness. (significance level *P*<0.05). Owing to non-normality of the rating scores from the emotion regulation task in the neutral condition (positively skewed), a Friedman’s ANOVA was applied to examine the main effect of condition on negative affect per group (*P*<0.05). *Post hoc* analyses were performed with a Wilcoxon signed-rank test (*P*<0.017, Bonferroni correction for three tests). To examine group differences on the rating scores of the emotion regulation task, Mann–Whitney *U*-tests were performed (*P*<0.017, Bonferroni correction for three tests).

The fMRI analyses were performed using Statistical Parametric Mapping (SPM8; www.fil.ion.ucl.ac.uk) running in Matlab7 (The MathWorks, Natrick, MA, USA). First, all images were checked for artifacts. Second, slice timing was applied and the functional images were spatially realigned, resliced, and coregistered to the anatomical scan. The anatomical images were segmented. Furthermore, the Diffeomorphic Anatomical Registration Through Exponentiated Lie algebra (DARTEL) approach was used to create a gray matter template based on the gray matter segmented images to enhance the accuracy of intersubject alignment. This template was used to normalize and affine transform the functional images to Montreal Neurological Institute (MNI) stereotactic space. A Gaussian kernel of 6 mm FWHM was applied to smooth the data. Head movement >3 mm in more than one direction resulted in exclusion of the data (one participant).

Thirteen task-related regressors were modeled with a boxcar function convolved with a hemodynamic response function. The regressors View and Relax were divided into View/Relax neutral and View/Relax negative. For Regulation, Linger, and Rating, separate regressors were made for Reappraise, Attend Negative, and Attend Neutral. In addition, the realignment parameters and the first derivatives thereof were entered as covariates to correct for possible effects related to head motion. Four contrasts were made for each participant: (1) View Negative versus View Neutral, to examine negative emotion processing; (2) View Neutral versus Fixation (e.g., constant), to examine whether findings in contrast 1 could be explained by differences in neutral image processing; (3) Reappraisal versus Attend Negative, to examine the neural correlates of cognitive reappraisal; and (4) Attend Negative versus Attend Neutral, to examine whether findings in contrast 3 could be explained by differences in neural correlates while attending negative pictures.

To examine task-related activation, one sample *t*-tests in healthy controls and UHR subjects were conducted separately. Sex and handedness were entered as covariates. Two-sample *t*-tests were performed to examine group differences on task-related activation for all four abovementioned contrasts, with sex and handedness as covariates. To limit possible false positives due to multiple comparisons, effects had to meet *P*<0.05 family-wise error corrected at cluster level to be considered statistically significant (initial height-threshold *P*<0.001). Because of specific hypotheses regarding the amygdala, a Small Volume Correction (SVC) was applied for this region.

## Results

### Demographic and behavioral results

One participant from the UHR group was excluded from the analyses because of poor data quality due to head motion. The final sample therefore consisted of 15 UHR participants and 16 healthy controls.

The behavioral data showed that UHR participants and healthy controls did not differ significantly on gender, age, education, handedness, reported use of suppression, and positive affect (see Table 1). However, UHR participants reported to use less reappraisal than controls and reported more negative affect before scanning (see Table 1). Variances between groups did not differ significantly, except for the PANAS negative score (F=13.4, *P*=0.001). Therefore, equal variances were not assumed (see Table 1).

On the emotion regulation task ratings, a main effect of condition was found in both the controls (*χ* ^2^(2)=32.0, *P*<0.001) and the UHR group (*χ* ^2^(2)=25.7, *P*<0.001). All participants rated the negative pictures as more negative compared with the neutral pictures (controls: *Z*=3.5, *P*<0.001; UHR: *Z*=3.4, *P*=0.001). Furthermore, all participants were capable of reducing their negative affect during reappraisal (controls: *Z*=−3.5, *P*<0.001; UHR: *Z*=−3.2, *P*=0.001) compared with attending negative pictures. No group differences between healthy controls and UHR participants were found on the ratings of negative affect, apart from a slightly higher rating of negative affect after attending neutral pictures in the UHR group (*P*=0.03, not reaching the multiple comparison threshold).

### Neuroimaging results

#### Main task effects

##### Emotional processing

The first 2 s of viewing a negative picture, compared with neutral, revealed higher activation in the control group in the bilateral middle temporal gyrus, retrosplenial cortex, bilateral fusiform gyrus, and right VLPFC (see Supplementary Table S1). Furthermore, the left (*P*_SVC_=0.02, *k*=14, *Z*=3.8, −22,−6,−18 (*x,y,z*)) and right (*P*_SVC_=0.01, *k*=19, *Z*=3.6, 26,0,−24 (*x,y,z*)) amygdalae were higher activated during negative picture viewing compared with neutral in the control group. UHR participants showed a similar pattern of activation (see Supplementary Table S1) and higher activation in the left amygdala (*P*_SVC_=0.01, *k*=36, *Z*=3.9, −26,2,−24 (*x,y,z*)) during negative picture viewing.

The 4 s of attending a negative picture, revealed higher activation in the bilateral inferior occipital gyrus and left calcarine sulcus in the control group (see Supplementary Table S1) in comparison with attending neutral pictures. UHR participants also activated the visual cortex while attending negative pictures, together with the DMPFC and the orbitofrontal cortex (see Supplementary Table S1).

##### Emotion regulation

Reappraising negative pictures compared with attending negative pictures resulted in increased activation in the VLPFC, the left middle and superior temporal gyrus, and in the DMPFC in the control group (see Supplementary Table S1). In the UHR group no significant activation differences were found in this contrast.

#### Group differences

##### Emotional processing

Viewing negative pictures, compared with viewing neutral pictures, resulted in higher activation in the posterior cingulate cortex (PCC) in the UHR group compared with the control group (see Table 2). This result was caused by less activation in the PCC during neutral picture viewing in the UHR group (see Table 2). No group differences were found for the contrast attending negative pictures versus attending neutral pictures.

##### Emotion regulation

**Figure 2:**
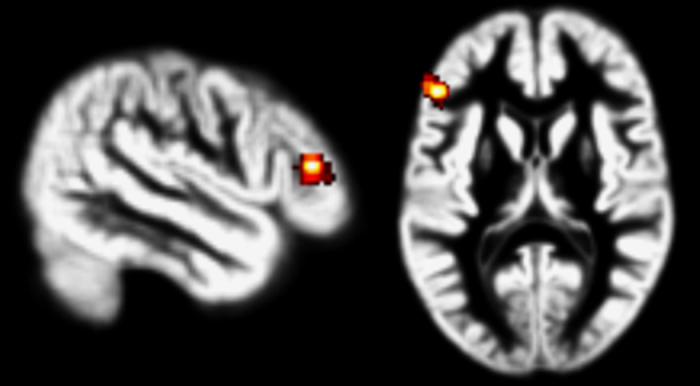
Higher activation in the left ventrolateral prefrontal cortex (VLPFC) in healthy controls compared with ultrahigh risk (UHR) subjects for the contrast reappraisal>attend negative. Results are displayed at *P*<0.001 with a *P*<0.05 FWE correction at the cluster level and overlaid on a normalized gray matter template based on the segmented T1 images of all participants.

During reappraisal, compared to attend negative, less activation in the left VLPFC was observed in the UHR group compared with controls (see Figure 2 and Table 2). When excluding the four UHR participants using antidepressants, this finding remained significant (*P*=0.03; *k*=112; *Z*=4.6; −44,26,12 (*x,y,z*)). Furthermore, this finding remained significant after controlling for cognitive alexithymia (see Supplementary 2). No activation differences during attend negative were found between the UHR and the control group.

## Discussion

The aim of the current study was to examine whether individuals at UHR for developing psychosis differ from healthy controls in brain activation during emotion regulation. The results revealed less activation of the left VLPFC in UHR individuals during reappraisal. Furthermore, UHR individuals reported less use of reappraisal in daily life (as measured with the ERQ) and showed higher rates of negative affect, compared with controls.

Reappraising negative pictures resulted in activation of the left temporal cortex, bilateral VLPFC, and bilateral DLPFC, and DMPFC in healthy controls. This activation pattern is consistent with previous neuroimaging studies.^16,37^ In line with our hypothesis, the UHR individuals activated the left VLPFC to a lesser extent during reappraisal compared with controls. The left VLPFC is consistently activated across reappraisal studies in healthy subjects^16,37^ and is specifically involved in the cognitive regulation of feelings.^38^ Lower activation in the VLPFC during reappraisal has previously been reported in patients with schizophrenia.^9^ The current results show that this lower level of activation is also present in UHR individuals.

Activation of the left VLPFC has been shown to be positively correlated with reappraisal success.^39^ This suggests that UHR individuals might be less successful in applying reappraisal, which has also been reported in patients with schizophrenia.^9^ However, during the task the UHR individuals were equally capable in reducing negative affect through reappraisal as the controls. Nevertheless, UHR individuals did report a tendency to use less reappraisal in daily life. This lower reported use of reappraisal has also been found in patients with schizophrenia,^10–12^ although not consistently.^13,14^ These inconsistent findings within patients with schizophrenia may indicate that only some types of schizophrenia patients experience difficulties with emotion regulation, which may also apply to UHR individuals. However, further research is required. One possible explanation for the nonsignificant group differences on negative affect after reappraisal during the task could be that UHR individuals were still capable of applying reappraisal in a structured laboratory setting, even though they could not fully recruit the relevant circuitry. Consequently, application of reappraisal might fail in more complex daily life situations, as reflected by the lower tendency to use reappraisal in daily life. Another possibility could be that the 4-point rating scale was not sensitive enough to pick up small group differences in negative affect reduction after reappraisal.

Furthermore, the results showed a higher level of negative affect in UHR individuals before the fMRI scan, which is typical for this group.^26^ Less reported use of reappraisal has been associated with higher levels of negative affect.^14,40^ This might indicate that difficulties with emotion regulation in UHR individuals result in more negative affect. More negative affect is suggested to precede psychotic episodes, presumably owing to emotion regulation difficulties.^5,7^ Our results support this hypothesis by revealing emotion regulation difficulties and more negative affect in the UHR-group. Notably, previous research has revealed associations between reappraisal difficulties and both lower social functioning^41^ and higher levels of psychotic symptoms.^14^ This suggests that emotion regulation difficulties, reflected by less VLPFC activation, might put individuals at increased risk for psychosis. Notably, in healthy college students with a slightly increased risk for developing psychosis (high psychosis proneness), higher activation in the VLPFC was found during reappraisal.^42^ The authors hypothesized that this higher activation might reflect a compensatory mechanism. This could indicate that there might be an inverted U-shape pattern of VLPFC activation in risk groups for psychosis during reappraisal. Groups with a slightly increased risk might still be capable of applying reappraisal through compensatory VLPFC activation, while groups at UHR might not show this compensation anymore, reflected by lower VLPFC activation. However, further research will be necessary to examine this hypothesis.

Viewing negative pictures revealed higher activation in emotion processing areas, such as the amygdala, middle temporal gyrus, and fusiform gyrus,^43^ in both control and UHR individuals. No differences between groups were found during viewing of negative emotions, except for higher PCC activation in the UHR group. This result was caused by lower PCC activation in the UHR group during neutral picture viewing. Previous research has shown that patients with schizophrenia show higher activation in the PCC in response to neutral faces.^44^ Hall *et al.* explained this higher activation by hypothesizing that patients with schizophrenia ascribe affective importance to neutral stimuli. However, in the current study the results revealed lower PCC activation in response to neutral stimuli in the UHR group. This difference is difficult to explain, but these results may indicate that the PCC also shows aberrant functioning in response to neutral pictures in UHR individuals. The only other fMRI-study examining the neural basis of negative emotion processing in people at UHR for psychosis found activation differences, only when examining interaction effects with age.^45^ Furthermore, neuroimaging studies on emotion processing in relatives of patients, with a slightly elevated genetic risk for psychosis, have produced equivocal results. Some reported increased^46,47^ or decreased activation,^48,49^ whereas others were unable to find activation differences during negative emotion processing.^50^ Therefore, further research is necessary to investigate the neural basis in individuals at (ultrahigh) risk for schizophrenia.

Several limitations of this study should be addressed. First, although we replicated the robust finding that reappraisal is associated with increased prefrontal activation, we did not observe a subsequent decrease of amygdala activation.^16^ A number of other studies also failed to find such a decrease.^51–53^ This has been attributed to the late cueing method, that we also applied (i.e., giving the instruction to reappraise after 2 s of stimuli presentation). This late cueing method was applied to allow participants to have a naturalistic response to the negative valence picture before regulation starts.^54^ However, this late cuing method might cause the amygdala to respond and habituate already before reappraisal starts.^54^ Future studies should investigate the frontal-limbic coupling in UHR individuals during reappraisal with an early cueing paradigm. Second, our aim was to examine whether individuals at UHR for psychosis show aberrant activations during emotion regulation. Owing to the small sample of the UHR group we were unable to examine the neural correlates specific to the transition towards psychosis. It is estimated that only about a third of these individuals will make the transition toward psychosis ^20^ and these estimated transition rates seem to decline over the last years.^55^ Therefore, it remains unclear whether these aberrant activations may predict the transition toward psychosis. Third, emotion regulation is a complex concept in which many different processes have a role. For example, reappraisal has been linked to neurocognitive functioning^56^ and previous research has suggested that cognitive emotion regulation difficulties in psychotic disorders may be more related to neurocognitive functioning.^15^ It is therefore possible that neurocognitive deficits in the UHR group are partly underlying the activation differences during reappraisal. Unfortunately, in the current study, neurocognitive functioning was not assessed. Although the control participants were matched for education level, possible differences in neurocognitive functioning might be related to the lower activation in the VLPFC. Fourth, the current study only focused on two emotion regulation strategies, reappraisal, and suppression (via the ERQ). However, there are other emotion regulation strategies, which have previously been associated with schizophrenia (e.g., see Rowland *et al.* ^15^). We therefore recommend future research to also examine other emotion regulation strategies, such as distraction and emotion-focused regulation in UHR individuals.

## Conclusions

To conclude, these results may indicate that emotion regulation difficulties, and associated reduction in activation of the VLPFC, are present in individuals at UHR for developing psychosis. These regulation difficulties could help explain the higher negative affect and lower social functioning that these individuals experience in daily life, which lead them to seek help. Therefore, it might be interesting for future studies to explore whether UHR individuals might benefit from emotion regulation training.

## Figures and Tables

**Table 1:** Mean, s.d. and group differences for demographic data, questionnaire data, and rating scores of the emotion regulation task and descriptives of medication status and psychotic symptoms

	*HC (*n*=16) Mean±s.d.*	*UHR (*n*=15) Mean±s.d.*	*Test statistic*		*Confidence interval (95%)*	
*Demographics*						
Gender (% male)	50%	53%	*χ* ^2^(1)=0.02	*P*=0.88	−0.32	0.38
Age (in years)	22.1±3.6	23.1±4.4	*t*(29)=−0.70	*P*=0.49	−4.0	2.0
Education^a^	5.4±0.6	5.2±09	*t*(29)=0.88	*P*=0.37	−0.31	0.79
Handedness (% right)	75%	80%	*χ* ^2^(1)=0.02	*P*=0.88	−0.26	0.34
						
*PANAS*						
Positive affect	33.4±5.4	30.0±7.7	*t*(29)=1.48	*P*=0.15	−1.33	8.34
Negative affect	12.4±2.0	22.3±6.7	*t*(16.3)=−5.5	*P*<0.001^b^^,^ c	−13.75	−6.16
						
*ERQ*						
Reappraisal	5.1±1.0	4.2±09	*t*(29)=2.74	*P*=0.01^b^	0.24	1.63
Suppression	4.5±1.7	4.1±1.2	*t*(29)=0.85	*P*=0.40	−0.62	1.52
						
*Rating scores*						
Attend neutral	1.1±02	1.3±04	*U*=64.0	*P*=0.03^d^		
Attend negative	2.4±05	2.5±07	*U*=108.5	*P*=0.65		
Reappraise	1.8±04	2.1±06	*U*=86.5	*P*=0.18		
						
*Current medication status (n)*						
Antipsychotics	0	0				
Antidepressants (SSRI)	0	4				
Methylphenidate	0	2				
Other medication	1	1				
						
*PANSS*						
Positive symptoms	NA	13.1±2.7				
Negative symptoms	NA	10.5±2.5				
General symptoms	NA	28.3±6.1				
						
*DSM diagnosis (n)*						
*MDD*		3				
Depressive disorder nos	NA	1				
Adjustment disorder	NA	4				
Personality disorder nos	NA	1				
OCD	NA	1				
Eating disorder nos	NA	1				
ADHD	NA	1				
PTSD	NA	1				
Learning disorder NOS	NA	1				
Unspecified mental disorder (non-psychotic)	NA	1				

Abbreviations: ADHD, Attention deficit Hyperactivity disorder; ERQ, Emotion regulation questionnaire; HC, healthy control; MDD, Major depressive disorder; NOS, Not otherwise specified; OCD, Obsessive Compulsive Disorder; PANAS, Positive and negative symptom scale; PANSS, Positive and Negative Syndrome Scale; PTSD, post-traumatic stress disorder; SSRI: Selective serotonin reuptake inhibitor; UHR, ultra-high risk.

a Education according to Verhage.^57^

b Significant at *P*<0.05.

c Equal variances not assumed.

d Not significant at the corrected *P*-value of *P*<0.017.

**Table 2:** Summary of significant brain activation differences between UHR individuals and controls during neutral image processing versus fixation, negative image processing versus neutral image processing, and reappraisal versus negative image processing

*Brain region*	*Hemisphere*	k* voxels*	*MNI coordinates*			Z
			x	y	z	
*View Neutral>Fixation*						
Controls>UHR						
Temporal pole	L	158	−36	22	−22	5.34
			−28	12	−24	4.11
Posterior cingulate gyrus	R/L	273	8	−50	28	4.23
			−4	−26	22	3.99
			0	−40	18	3.80
						
*View Negative >View Neutral*						
UHR >Controls						
Posterior cingulate gyrus	R/L	144	−6	−52	30	3.73
			8	−50	28	3.67
			0	−42	32	3.64
						
*Reappraise >Attend Negative*						
Controls >UHR						
Inferior frontal gyrus triangular part	L	168	−46	26	12	4.76

Abbreviation: UHR, ultrahigh risk group.
