# Supplementary material for: Lower prefrontal activation during emotion regulation in subjects at ultrahigh risk for psychosis: an fMRI-study
Source: NPJ Schizophr. 2015 Sep 23;1:15026–. doi: 10.1038/npjschz.2015.26 (PMC4849453; doi:10.1038/npjschz.2015.26)
Supplement: Supplementary Table S1 [file npjschz201526-s1.doc]

**Supplementary material**

**Supplementary table S1**

Main effects of negative emotion processing and reappraisal on BOLD responses

|  |  |  | MNI coordinates | | |  |
| --- | --- | --- | --- | --- | --- | --- |
|  | Hemisphere | k voxels | x | y | z | *Z* |
|  |  |  |  |  |  |  |
| *View Negative > View Neutral* |  |  |  |  |  |  |
|  |  |  |  |  |  |  |
| *HC only* |  |  |  |  |  |  |
| Middle temporal gyrus | R | 1434 | 44 | -60 | 8 | 6.15 |
|  |  |  | 52 | -66 | 4 | 5.77 |
|  |  |  | 48 | -72 | -2 | 5.77 |
| Middle temporal gyrus | L | 1261 | -44 | -64 | 6 | 5.76 |
|  |  |  | -48 | -72 | 8 | 5.65 |
|  |  |  | -48 | -76 | 0 | 5.43 |
| Retrosplenial cortex | R/L | 572 | -6 | -56 | 12 | 5.52 |
|  |  |  | 8 | -52 | 8 | 4.98 |
|  |  |  | 18 | -56 | 18 | 3.55 |
| Fusiform gyrus | L | 114 | -26 | -36 | -18 | 5.48 |
| Ventrolateral prefrontal cortex | R | 441 | 46 | 10 | 28 | 5.45 |
|  |  |  | 32 | 8 | 30 | 4.47 |
|  |  |  | 48 | 22 | 24 | 3.66 |
| Fusiform gyrus | R | 161 | 42 | -46 | -20 | 4.85 |
|  |  |  | 38 | -64 | -18 | 3.23 |
| Brainstem | R/L | 139 | -2 | -28 | -6 | 4.50 |
|  |  |  | 10 | -26 | -18 | 4.01 |
| Brainstem | R/L | 165 | -4 | -18 | -18 | 4.28 |
|  |  |  | 8 | -16 | -14 | 4.00 |
|  |  |  | 6 | -8 | -8 | 3.98 |
| Middle temporal gyrus | R | 147 | 54 | -6 | -18 | 4.13 |
|  |  |  | 48 | -12 | -14 | 3.51 |
|  |  |  | 50 | 2 | -26 | 3.48 |
|  |  |  |  |  |  |  |
| *UHR only* |  |  |  |  |  |  |
| Middle temporal gyrus | R | 1813 | 44 | -58 | 8 | 6.25 |
|  |  |  | 54 | -68 | 4 | 6.24 |
|  |  |  | 46 | -74 | -2 | 4.99 |
| Middle temporal gyrus | L | 1715 | -48 | -72 | 8 | 5.59 |
|  |  |  | -40 | -60 | 12 | 5.52 |
|  |  |  | -38 | -62 | 26 | 4.77 |
| Thalamus | R/L | 995 | 8 | -26 | -8 | 5.17 |
|  |  |  | -4 | 0 | 8 | 4.98 |
|  |  |  | 0 | 24 | 0 | 4.60 |
| Ventrolateral prefrontal cortex | L | 192 | -42 | 24 | -16 | 5.17 |
|  |  |  | -46 | 28 | 4 | 4.91 |
|  |  |  | -42 | 18 | -26 | 4.60 |
| Fusiform gyrus | R | 129 | 40 | -50 | -20 | 5.15 |
| Retrosplenial cortex | R/L | 1684 | -6 | -56 | 26 | 5.12 |
|  |  |  | -2 | -40 | 34 | 5.04 |
|  |  |  | 8 | -52 | 28 | 4.95 |
| Dorsomedial prefrontal cortex | R/L | 699 | -4 | 60 | 30 | 4.80 |
|  |  |  | -2 | 50 | 38 | 4.18 |
|  |  |  | 6 | 50 | 32 | 4.04 |
| Ventrolateral prefrontal cortex | R | 187 | 50 | 18 | 22 | 4.38 |
|  |  |  | 42 | 24 | 22 | 3.47 |
|  |  |  | 48 | 22 | 12 | 3.61 |
| Middle temporal gyrus | L | 160 | -58 | -2 | -18 | 4.33 |
|  |  |  | -46 | 6 | -30 | 3.91 |
|  |  |  | -50 | -10 | -22 | 3.85 |
| Cerebellum | R/L | 157 | 4 | -58 | -48 | 4.04 |
|  |  |  | 14 | -52 | -44 | 3.79 |
|  |  |  | -10 | -54 | -46 | 3.78 |
|  |  |  |  |  |  |  |
| *Attend negative > Attend neutral* |  |  |  |  |  |  |
|  |  |  |  |  |  |  |
| *HC only* |  |  |  |  |  |  |
| Inferior occipital gyrus | R | 2533 | 40 | -74 | -2 | 6.74 |
|  |  |  | 48 | -74 | -8 | 6.59 |
|  |  |  | 24 | -92 | 4 | 5.04 |
| Inferior occipital gyrus | L | 1339 | -44 | -78 | -12 | 5.72 |
|  |  |  | -48 | -74 | 2 | 5.39 |
|  |  |  | -38 | -82 | -2 | 5.31 |
| Calcarine sulcus | L | 131 | -6 | -92 | 4 | 4.14 |
|  |  |  | -14 | -90 | -2 | 4.28 |
|  |  |  |  |  |  |  |
| *UHR only* |  |  |  |  |  |  |
| Inferior occipital gyrus | R | 2564 | 48 | -74 | -8 | 5.86 |
|  |  |  | 42 | -74 | -2 | 5.47 |
|  |  |  | 32 | -90 | -8 | 5.09 |
| Inferior occipital gyrus | L | 1645 | -48 | -74 | 2 | 5.10 |
|  |  |  | -36 | -84 | -16 | 4.71 |
|  |  |  | -42 | -78 | -12 | 4.63 |
| Precuneus | R/L | 474 | 0 | -56 | 52 | 4.85 |
|  |  |  | 8 | -66 | 50 | 4.07 |
|  |  |  | -2 | -64 | 50 | 3.96 |
| Cerebellum | R | 240 | 8 | -82 | -30 | 4.56 |
|  |  |  | 6 | -82 | -20 | 3.78 |
|  |  |  | 4 | -78 | -40 | 3.73 |
| Dorsomedial prefrontal cortex | R/L | 114 | 2 | 26 | 40 | 4.15 |
|  |  |  | -6 | 14 | 42 | 3.99 |
| Orbitofrontal cortex | L | 223 | -42 | 50 | -2 | 3.80 |
|  |  |  | -40 | 56 | 6 | 3.77 |
|  |  |  | -26 | 60 | 16 | 3.74 |
|  |  |  |  |  |  |  |
| *Reappraise > Attend negative* |  |  |  |  |  |  |
|  |  |  |  |  |  |  |
| *HC only* |  |  |  |  |  |  |
| Inferior frontal gyrus triangular part | L | 1397 | -50 | 24 | 8 | 5.49 |
|  |  |  | -44 | 30 | -8 | 5.38 |
|  |  |  | -52 | 20 | -2 | 4.79 |
| Middle temporal gyrus | L | 195 | -50 | 4 | -28 | 5.25 |
|  |  |  | -46 | 4 | -38 | 5.17 |
|  |  |  | -52 | 8 | -16 | 3.60 |
| Dorsomedial prefrontal cortex | R/L | 1190 | -4 | 36 | 34 | 4.74 |
|  |  |  | -14 | 14 | 50 | 4.64 |
|  |  |  | 12 | 24 | 52 | 4.49 |
| Middle temporal gyrus | L | 129 | -62 | -38 | -6 | 4.48 |
|  |  |  | -54 | -32 | -10 | 3.51 |
| Superior temporal gyrus | L | 471 | -56 | -46 | 20 | 4.40 |
|  |  |  | -44 | -60 | 22 | 4.21 |
|  |  |  | -52 | -60 | 34 | 4.19 |
| Inferior frontal gyrus operculum | R | 170 | 60 | 18 | 8 | 3.77 |
|  |  |  | 46 | 20 | -2 | 3.76 |
|  |  |  | 52 | 32 | -8 | 3.32 |
|  |  |  |  |  |  |  |
| *UHR only* |  |  |  |  |  |  |
| No significant results |  |  |  |  |  |  |
|  |  |  |  |  |  |  |

*Abbreviations: HC = healthy controls; L = left; MNI = Montreal Neurological Institute; R = right; UHR = ultra-high risk group*
