# Supplementary material for: Lower prefrontal activation during emotion regulation in subjects at ultrahigh risk for psychosis: an fMRI-study
Source: NPJ Schizophr. 2015 Sep 23;1:15026–. doi: 10.1038/npjschz.2015.26 (PMC4849453; doi:10.1038/npjschz.2015.26)
Supplement: Supplementary Information [file npjschz201526-s2.doc]

**Supplement 2**

To examine whether differences between the groups in the capability of introspection and identifying emotions could have influenced the fMRI results, we examined cognitive alexithymia as measured with the Bermond-Vorst Alexithymia Questionnaire (BVAQ; Vorst and Bermond, 2001). Cognitive alexithymia represents the ability to identify, verbalize and analyze feelings (Bermond et al., 2007) and is closely related to introspection.

The average levels of cognitive alexithymia in the UHR group and control group are presented in supplementary table 2.1. The Mann-Whitney U tests revealed that the UHR group had significantly higher levels of cognitive alexithymia compared to controls. This data is part of a recently accepted paper *(Van der Velde et al., accepted).* Because of these significant differences, the cognitive alexithymia scale was included as an additional covariate to the fMRI analysis to examine whether this would influence the findings. The results showed that the lower activation in the inferior frontal gyrus remained significant even after controlling for alexithymia. Furthermore, two additional clusters survived the FWE cluster correction threshold after correcting for alexithymia, namely the supplementary motor area and the right inferior frontal gyrus (Table 2.2).

**Table 2.1**Group differences on cognitive alexithymia between controls and UHR subjects

|  | Controls (n=16)  Mean ± SD | UHR (n=15)  Mean ± SD | Test statistic |
| --- | --- | --- | --- |
| Cognitive alexithymia | 56.6 ± 14.7 | 71.5 ± 12.5 | U=52, p=.007* |
| Identifying | 16.0 ± 3.8 | 23.1 ± 5.7 | U=38, p=.001* |
| Verbalizing | 22.8 ± 7.2 | 30.1 ± 5.8 | U=55, p=.01* |
| Analyzing | 17.9 ± 6.7 | 18.3 ± 5.3 | U=109, p=.66 |

*Abbreviations*: SD: standard deviation; UHR: ultra-high risk

**Table 2.2**

Summary of significant brain activation differences between UHR individuals and controls during reappraisal versus negative image processing including cognitive alexithymia as a covariate.

|  |  |  | MNI coordinates | | |  |
| --- | --- | --- | --- | --- | --- | --- |
| Brain region | Hemisphere | k voxels | x | y | z | *Z* |
|  |  |  |  |  |  |  |
| *Controls > UHR* |  |  |  |  |  |  |
| Supplementary motor area | R/L | 148 | 10 | -6 | 62 | 4.41 |
|  |  |  | -2 | -6 | 64 | 4.30 |
|  |  |  | -10 | -6 | 58 | 3.79 |
| Inferior frontal gyrus | R | 113 | 44 | 10 | 6 | 4.09 |
|  |  |  | 44 | 0 | 10 | 3.68 |
| Inferior frontal gyrus | L | 117 | -46 | 26 | 14 | 4.03 |

*Abbreviations: UHR: ultra-high risk group*

**References**

Bermond, B., Clayton, K., Liberova, A., Luminet, O., Maruszweski, T., Ricci Bitti, P.E., Rime, B., Vorst, H., Wagner, H., Wicherts, J. (2007). A cognitive and an affective dimension of alexithymia in six languages and seven populations. Cognitive emotion, 21, 1125-1136.

Van der Velde, J., Swart, M., van Rijn, S., van der Meer, L., Wunderink, L., Wiersma, D., Krabbendam, L., Bruggeman, R., Aleman, A. Cognitive alexithymia is associated with the degree of risk for psychosis. PLoS One, accepted

Vorst, H.C.M., Bermond, B. (2001). Validity and reliability of the Bermond-Vorst Alexithymia Questionnaire, Per. Individ. Dif., 3, 413-434.
